# The Cement Prosthesis-Like Spacer: An Intermediate Halt on the Road to Healing

**DOI:** 10.1155/2013/763434

**Published:** 2013-09-30

**Authors:** Sufian S. Ahmad, Kim Huber, Dimitrios S. Evangelopoulos, Barbara Kleer, Hendrik Kohlhof, Michael Schär, Stefan Eggli, Sandro Kohl

**Affiliations:** Department of Orthopedic Surgery and Traumatology, University of Bern, Switzerland

## Abstract

*Background*. Periprosthetic infections remain a devastating problem in the field of joint arthroplasty. In the following study, the results of a two-stage treatment protocol for chronic periprosthetic infections using an intraoperatively molded cement prosthesis-like spacer (CPLS) are presented. *Methods*. Seventy-five patients with chronically infected knee prosthesis received a two-stage revision procedure with the newly developed CPLS between June 2006 and June 2011. Based on the microorganism involved, patients were grouped into either easy to treat (ETT) or difficult to treat (DTT) and treated accordingly. Range of motion (ROM) and the knee society score (KSS) were utilized for functional assessment. *Results*. Mean duration of the CPLS implant in the DTT group was 3.6 months (range 3–5 months) and in the ETT group 1.3 months (range 0.7–2.5 months). Reinfection rates of the final prosthesis were 9.6% in the ETT and 8.3% in the DTT group with no significant difference between both groups regarding ROM or KSS (*P* = 0.87, 0.64, resp.). *Conclusion*. The results show that ETT patients do not necessitate the same treatment protocol as DTT patients to achieve the same goal, emphasizing the need to differentiate between therapeutic regimes. We also highlight the feasibility of CLPS in two-stage protocols.

## 1. Introduction

 Osteoarthritis is nowadays a major cause of disability in adults with a growing trend. Alone knee osteoarthritis has a prevalence of over 30% amongst a population aged ≥60 years; this expresses the dimensions of the problem [1, 2].

Total knee arthroplasty (TKA) maintains its position as a major treatment option for knee osteoarthritis [3–5]. A feared complication of KA is infection of the prosthetic implant [6–8]. The reason for such concern is the substantial increase in morbidity and health care expenditure [9].

At least one of the following criteria has to be fulfilled to set the diagnosis of a prosthetic infection: growth of one microorganism species in two or more cultures of synovial fluid or periprosthetic tissue, purulence of the synovial fluid or macroscopic changes at the site of the implant, acute inflammation on histopathological examination of periprosthetic tissue, or presence of a sinus tract communicating with the prosthesis [10–12]. 

The gold standard for treating chronic periprosthetic infection is based on a two-stage protocol, including initial explantation of the infected components, adequate debridement, and antibiotic cement spacer prostheses implantation with systemic antibiotic therapy followed by secondary TKA once the optimal condition is achieved [13, 14]. The antimicrobial-impregnated spacer utilized in this process allows for maintenance of limb length, partial mobility during the recovery process, and infection control rates of 91% to 100% [15, 16]. Initially, cement spacers were static, therefore not providing sufficient range of motion (ROM); bone loss, soft tissue contracture, and increased scar tissue formation as a result have been mentioned [17–19]. The attempt to achieve a degree of ROM using dynamic cement on cement spacers with a joint geometry gained interest as a possible solution for the problems associated with static spacers [18, 19].

The use of either premolded spacers, intraoperative handcrafting by the surgeon, or intraoperative molding using standard predesigned moulds has been described in the literature [20–22].

In this paper we verify the safety and efficacy of an intraoperatively produced custom made polymethyl methacrylate (PMMA) cement prostheses like spacer (CPLS) for a two-stage revision protocol of infected total KA.

## 2. Materials and Methods

Two molds were produced using a computerized numerical-control sinking machine (DMU 70eV-process) based on the design of the balanSys knee system (size B; D) (Mathys AG, Bettlach, Switzerland). The molds for the femoral spacer consisted of 3 components and the tibial of 2 components made of 100% Teflon). These molds were utilized intraoperatively to produce the spacer in its wanted shape.

The first surgical step involved explantation the prosthetic components and extensive debridement of the infected region, and biopsies were taken during the process for microbiological culture and histological examination. CPLS was finally performed. Initially, the components' appropriate sizes were assessed by means of conventional anterior posterior and lateral knee radiographs. The parts of the femoral mould were mounted, and the mould was filled with cement by hand. Due to cement expansion, the increase in pressure inside the closed mould created a smooth surface on the final cement spacer. After polymerization, the screws which interlink the mould were opened, and the femoral component was easily removed. The femoral component was implanted first with a small portion of additional cement. The distance between the tibia and the femur was measured in neutral position and the tibial component has filled with PMMA cement according to the distance measured. After polymerization, the tibial part of the spacer was removed from the mould, and the mounting cement on the posterior and lateral side of the spacer was removed with a Luer pincer. The tibial component was then implanted with a small portion of cement. The stability and range of motion were tested, and the wound was closed.

For all PMMA spacers, an antibiotic loaded cement was applied: PALACOSR + G 40 (Heraeus Medical GmbH, Wehrheim, Germany), containing 0.5 g of Gentamycin. The system permits the incorporation of different antibiotics into the PMMA spacer according to the antibiogram obtained by the initial puncture.  Figure 2 shows the molds used intraoperatively, Figure 3 shows X-ray view of the implanted CPLS and Figure 4 shows an intraoperative view of an implanted CPLS immediately before revision TKA.

Seventy-five patients with chronically infected TKA received a two-stage revision procedure with the newly developed CPLS between June 2006 and June 2011 (mean age 67.5 years, range 57–85 years). However, two different protocols were considered according to the microorganism involved and treatment response (Figure 5). Patients infected with multidrug-resistant microorganisms, gram negative microorganisms, enterococcus species, or polymicrobial infections were considered *difficult to treat (DTT) (8)*, whereas patients infected with other microorganisms were considered *easy to treat (ETT).* All patients underwent joint aspiration for microbiological examination prior to surgery. 

In the DTT (*n* = 13) group, a CPLS was implanted for 12 weeks, during which systemic antibiotics were administered, an open biopsy performed after a two-week antibiotic-free interval to confirm absence of microorganism growth before performing the revision TKA and finally continuing the systemic antibiotic therapy for 3 months.

In the ETT (*n* = 62) group, a CPLS was implanted for 4–6 weeks, during which systemic antibiotics were administered, and finally the revision TKA was performed after normalization of inflammatory markers and optimization of the soft tissue condition. Otherwise the patient was considered DTT and treated according to protocol. Antibiotic treatment was discontinued after the second stage TKA in the ETT group.

Knee society score (KSS) [23] and range of motion (ROM) were used for functional assessment at the time of spacer implantation and at one-year followup after TKA re-implantation. Results were compared using the students *t*-test, and *P* ≤ 0.05 was considered statistically significant.

## 3. Results

Twenty-one ETT patients (27.9%) were infected with *Staphylococcus aureus*, nineteen (25.6%) with *coagulase-negative staphylococci*, nine (11.6%) with *alpha-hemolytic streptococci*, four (4.7%) with *beta-hemolytic streptococci*, two (2.3%) with *gram-positive rods*, two (2.3%) with *methicilin resistant staphylococcus aureus,* and five (7%) with an unknown microorganism.

**Figure 1:**
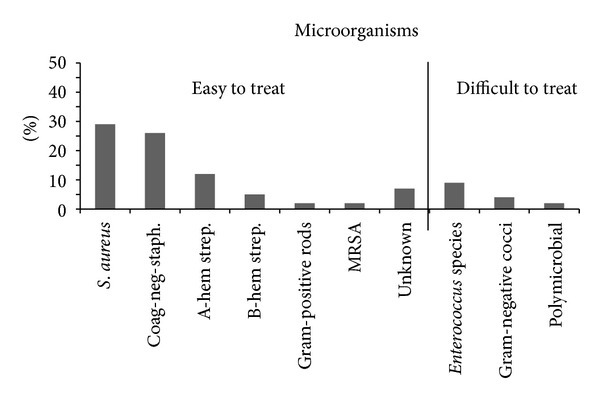
The percentage abundance of the encountered organisms in the cohort of this study. Coag-neg-staph: coagulase-negative staphylococci; A-hem strep: alpha-hemolytic streptococci. B-hem strep. Beta-hemolytic streptococci.

**Figure 2:**
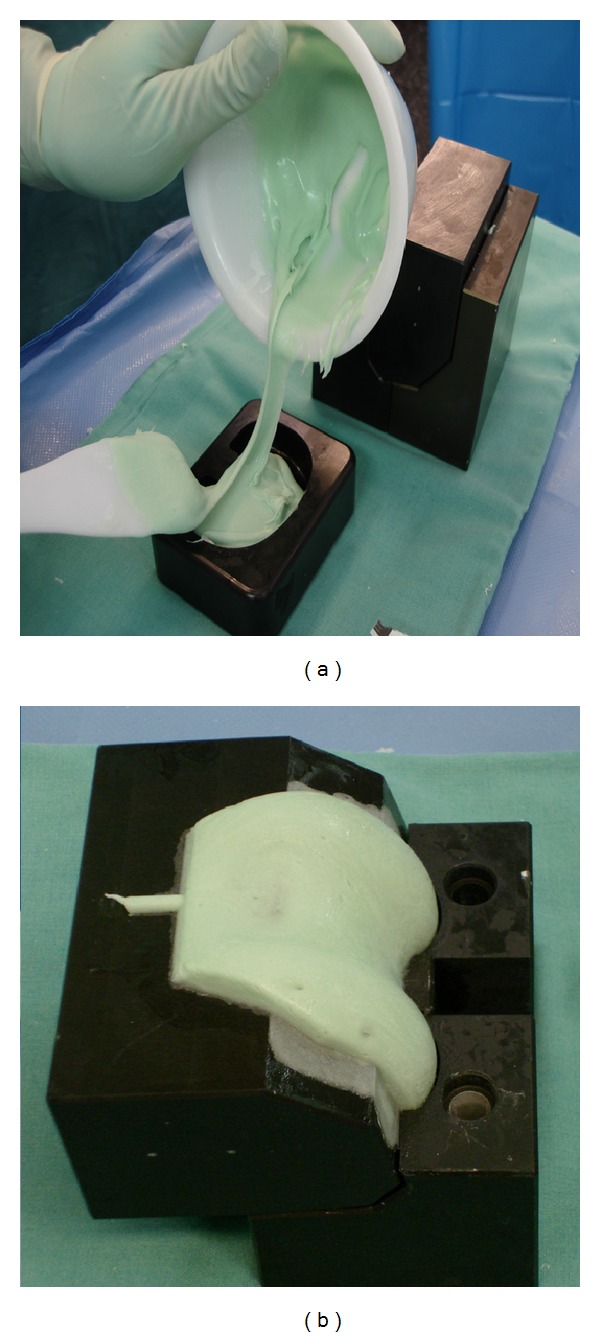
(a) Tibial mold, (b) femoral mold used intra-operatively.

**Figure 3:**
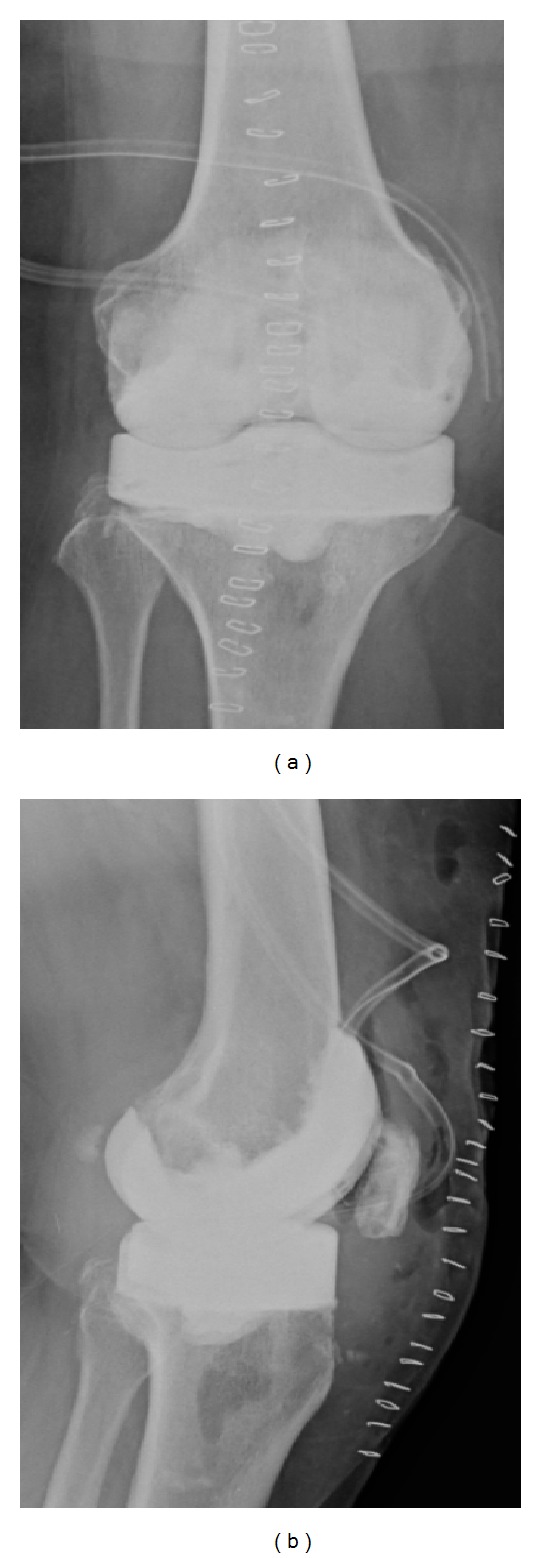
*X-ray images showing* (a) anterior posterior view with the CLPS implanted and (b) lateral view with the CLPS implanted.

Seven DTT patients (9.3%) were infected with *enterococcus* species, three (4%) were infected with *gram-negative cocci,* and two (2.3%) had a polymicrobial infection (Figure 1).

The knee patient database of our department was utilized for retrieval of data. The mean duration of the CPLS implant in the DTT group was 3.6 months (range 3–5 months) and in the ETT group was 1.3 months (range 0.7–2.5 months). With the CPLS *in situ*, all patients were mobilized on crutches till final revision, and with a maximum weight of 15–20 kg, full range of motion was allowed. Squeaking was reported by all patients during the first 2 weeks of CPLS implantation, and no pain was associated. CPLS components were stable upon X-ray followup 2 months after implantation in all patients. The mean range of ROM with CPLS generally was 103° (range 75°–130°): DTT group was 104° (range 77°–130°) was ETT 102° (range 75°–130°), and no significant difference between both groups (*P* = 0.87). The mean KSS was 84.4 (range 71–93): DTT group was 85.1 (range 71–91), ETT group 84.2 (range 73–93), with no significant difference between both groups (*P* = 0.64). 

Initial mean C-reactive protein (CRP) value immediately before explanation of the prosthesis was 150.1 mg/L (range 98 mg/L–235 mg/L): DTT group, mean 144.2 mg/L (range 114–235 mg/L), ETT group, mean 153.2 mg/L (range 98–222 mg/L), with no significant difference between both groups (*P* = 0.471). The mean CRP value immediately before performing the second stage TKA procedure was 8.87 mg/L (range 3–18 mg/L): DTT group, mean 9.4 mg/L (range 5–18 mg/L), ETT group, mean 8.1 mg/L (range 3–16 mg/L), with no significant difference between both groups (*P* = 0.152).

The mean follow-up interval after the final revision arthroplasty was 4.3 years (range 2–7 years).

The mean ROM with the final prosthesis generally was 115° (range 90°–125°): DTT group, mean 112° (range 90°–125°) and ETT group, mean 117° (range 82°–130°), with no significant difference between both groups (*P* = 0.76). The mean KSS was generally 89.5 (range 74–95): DTT group, mean 88.4 (range 76–93), ETT group, mean 90.2 (range 74–95), with no significant difference between both groups (*P* = 0.354).

In the ETT group, 6 reinfections (9.6%) of the final prosthesis occurred, 3 *Staphylococcus aureus, 2 Streptococci* and* 1 coagulase-negative staphylococcus during the first 6 months*. Two of these were persistent infections during the first 6 weeks requiring a divert of treatment protocol from ETT to DTT, one was a reinfection within the first 6 months requiring a divert to a DTT treatment protocol and three required a divert during the remaining follow-up interval.

In the DTT group one reinfection (8.3%) with *gram negative cocci* occurred 3.5 years after revision ending up in arthrodesis.

## 4. Discussion

Due to the devastating problem of chronic joint infections, work on the development of new strategies and material to tackle the problem is necessary. 

The one-stage revision arthroplasty is widely spread and maintains its place as treatment standard in many centers [24–26]. However, a review article published recently by Romano et al. showed that two-stage procedures provide benefit over one-stage procedures regarding reinfection rates, and that far more two-stage procedures are being reported in the literature showing the increasing popularity of two-stage procedures [27]. The eradication rate of 90.7% achieved in our 75 patient of two-stage series was higher than the literature average of 81.9 for one-stage procedures and close to the literature average of 91.2 for two-stage procedures using articulating spacers [27].

**Figure 4:**
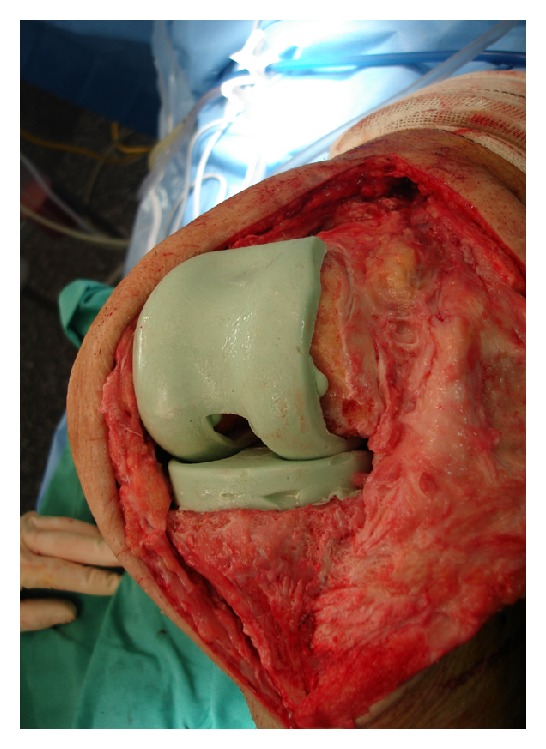
An intraoperative view of an implanted CPLS immediately before revision TKA.

**Figure 5:**
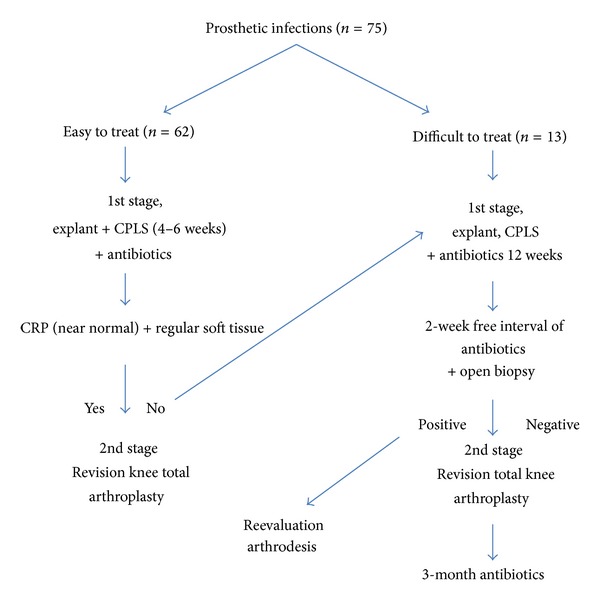
Protocol upon which the treatment strategy was based.

In this study, we present a therapeutic plan for peri-prosthetic infections based on the microorganism involved (Figure 4). Zimmerli et al. first described the term difficult to treat DTT prosthetic infections in association with the microorganisms mentioned above [8]. According to Zimmerli, we differentiated between ETT and DTT patients and used two different therapeutic protocols (Figure 4). The duration of treatment for ETT patients was significantly less than ETT patients (1.3 months versus 3.6 moths, resp.). Same for the antibiotic therapy that was discontinued immediately after revision TKA in ETT patients and continued for three months in DTT patients. The results did not show any significant difference in outcome regarding reinfection rates of the final prosthesis, ROM, or KSS scores between both groups. 

The insertion of an intraoperative moulded PMMA articulating spacer presents surgical advantages and apparently is associated with less reinfections than static spacers [27]. We noted that the application of such a spacer resulted in considerably less scar tissue formation, thus facilitating surgical exposure during the second intervention. The fact that less scar tissue removal had to be performed facilitated joint exposure avoiding complex soft tissue procedures, thus resulting in shorter operation time and easier postoperative rehabilitation.

In conclusion, the results show that ETT patients do not necsseate the same treatment protocol as DTT patients to achieve the same goal, emphasizing the need to differentiate between therapeutic regimes. We also highlight the feasibility of CLPS in two-stage protocols.
